# Mid-life serum Vitamin D concentrations were associated with incident dementia but not late-life neuropsychological performance in the Atherosclerosis Risk in Communities (ARIC) Study

**DOI:** 10.1186/s12883-019-1483-3

**Published:** 2019-10-22

**Authors:** Oluwaseun E. Fashanu, Di Zhao, Andrea L. C. Schneider, Andreea M. Rawlings, A. Richey Sharrett, Pamela L. Lutsey, Rebecca F. Gottesman, Alden L. Gross, Eliseo Guallar, Alvaro Alonso, Thomas H. Mosley, Erin D. Michos

**Affiliations:** 1 Ciccarone Center for the Prevention of Cardiovascular Disease, Johns Hopkins University School of Medicine, Blalock 524-B, 600 N. Wolfe Street, Baltimore, MD 21287 USA; 2 Department of Medicine, Saint Agnes Hospital, Baltimore, MD USA; 3 Department of Epidemiology, Johns Hopkins Bloomberg School of Public Health, Baltimore, MD USA; 4 Department of Neurology, Johns Hopkins University School of Medicine, Baltimore, MD USA; 5 The School of Biological and Population Health Sciences, Oregon State University, Corvallis, OR USA; 6 Division of Epidemiology and Community Health, University of Minnesota School of Public Health, Minneapolis, MN USA; 7 Center on Aging and Health, Johns Hopkins University, Baltimore, MD USA; 8 Department of Epidemiology, Rollins School of Public Health, Emory University, Atlanta, GA USA; 9 Department of Medicine, University of Mississippi Medical Center, Jackson, MS USA

**Keywords:** Vitamin D, Cognitive function, Cerebrovascular disease, Vascular risk factors

## Abstract

**Background:**

Activated Vitamin D has anti-inflammatory properties and adequate 25-hydroxyvitamin D [25(OH)D] concentrations may be important for neurocognitive function and protection against neurologic injury. We examined whether mid-life 25(OH) D concentrations were associated with later-life performance on neuropsychological testing, functional ability, depressive symptoms, and incident dementia.

**Methods:**

We studied 13,039 white and black ARIC participants who had serum 25(OH) D measured mid-life at visit 2 (1990–1992). Over the next ~ 20 years through visit 5 (2011–2013), participants underwent 3 additional in-person visits, annual telephone calls, and hospitalization surveillance. An extensive battery of neuropsychological outcomes were assessed at visit 5 using standardized protocols. Incident dementia was ascertained through a formal algorithm that included data from in-person cognitive testing, telephone interviews, hospital discharge codes, and death certificate codes. Diagnoses of dementia were adjudicated by expert clinician committee. For the primary cognitive analyses, we imputed for missing covariates and outcomes and used linear regression to evaluate non-concurrent cross-sectional associations of mid-life 25(OH) D (visit 2) with late-life neuropsychological outcomes (visit 5). We also used Cox regression models to examine associations of mid-life 25(OH) D and incident dementia.

**Results:**

In mid-life, the mean (SD) age of participants was 57 (6) years, 57% were women, and 24% black. Mean (SD) 25(OH) D was 24.3 (8.6) ng/mL; 33% had deficient (< 20 ng/mL), 44% intermediate (20- < 30 ng/mL), and 23% sufficient (≥30 ng/mL) 25(OH) D concentrations. Association between mid-life 25(OH) D and late-life performance on neuropsychological testing were mostly null. There was no significant association with functional ability or depressive symptoms. Results were similar in a sensitivity analysis using complete-case data (no imputation). However, after a median follow-up of 20 years, low 25(OH) D concentrations were associated with increased risk for incident dementia (*p* = 0.01 for trend across categories), with HR of 1.26 (95% CI 1.06, 1.49) for participants with deficient 25(OH) D, compared to sufficient concentrations.

**Conclusion:**

In this community cohort, mid-life serum 25(OH) D concentrations were associated with incident dementia but not with performance on neuropsychological testing, functional ability, or depressive symptoms, 20 years later. Whether serum 25(OH) D concentrations are causally related to dementia or confounded by poorer health status remains uncertain.

**Trial registration:**

Registered on clinicaltrials.gov NCT00005131.

## Introduction

Vitamin D in its activated form [1,25-dihydroxyvitamin D] may be important for cognitive functioning and protective against neurovascular injury [1, 2]. Vitamin D receptors are located in the cortex and hippocampus, areas of the brain important for cognitive functioning, and vitamin D receptor downregulation in these areas has been associated with Alzheimer’s disease [1]. Therefore, adequate serum concentrations of 25-hydroxyvitamin D [25(OH)D], the primary circulating form of vitamin D, in mid-life may help prevent cognitive decline, Alzheimer’s disease and vascular dementia in later-life. However, prior research on the associations between 25(OH) D and cognitive functioning have provided mixed results [3–11]. Nevertheless, a recent systematic review and meta-analysis of five cohort studies provided some evidence towards a positive and significant association of low 25(OH) D with dementia risk [5].

Additionally, vitamin D receptor gene polymorphisms have been associated with depressive symptoms [12]. Low 25(OH) D concentrations have also been associated with muscle weakness [13], reduced physical performance [14], frailty [15], and falls [16], but interventional trials of vitamin D supplementation on functional outcomes have shown no benefit or have been inconclusive [17–19]. The identification of easily modifiable risk factors in the regulation of mood and physical function in the elderly is of great importance as these affect the quality of life [20, 21].

In sum, the relationship of 25(OH) D concentrations with cognitive function, depression, and physical functioning have been inconclusive to date. Cross-sectional studies conducted in the elderly of the associations of 25(OH) D with neuropsychological outcomes may be limited by reverse causation. To further address this knowledge gap, we therefore examined associations between mid-life 25(OH) D concentrations and late-life performance on a comprehensive battery of neuropsychological, functional, and depression testing, and the association with incident dementia, in the Atherosclerosis Risk in Communities (ARIC) cohort.

## Methods

### Study population

The ARIC study is an ongoing community-based cohort which in 1987–1989 enrolled 15,792 participants, aged 45–65 years, from four U.S. communities, as previously described [22]. After baseline, participants attended up to five additional in-person study visits (which included cognitive testing at 4 of these visits), and they were also followed by semi-annual telephone interviews and active surveillance of community hospitals. ARIC was approved by the institutional review boards of each participating institution, and all participants provided written informed consent at each visit.

**Fig. 1:**
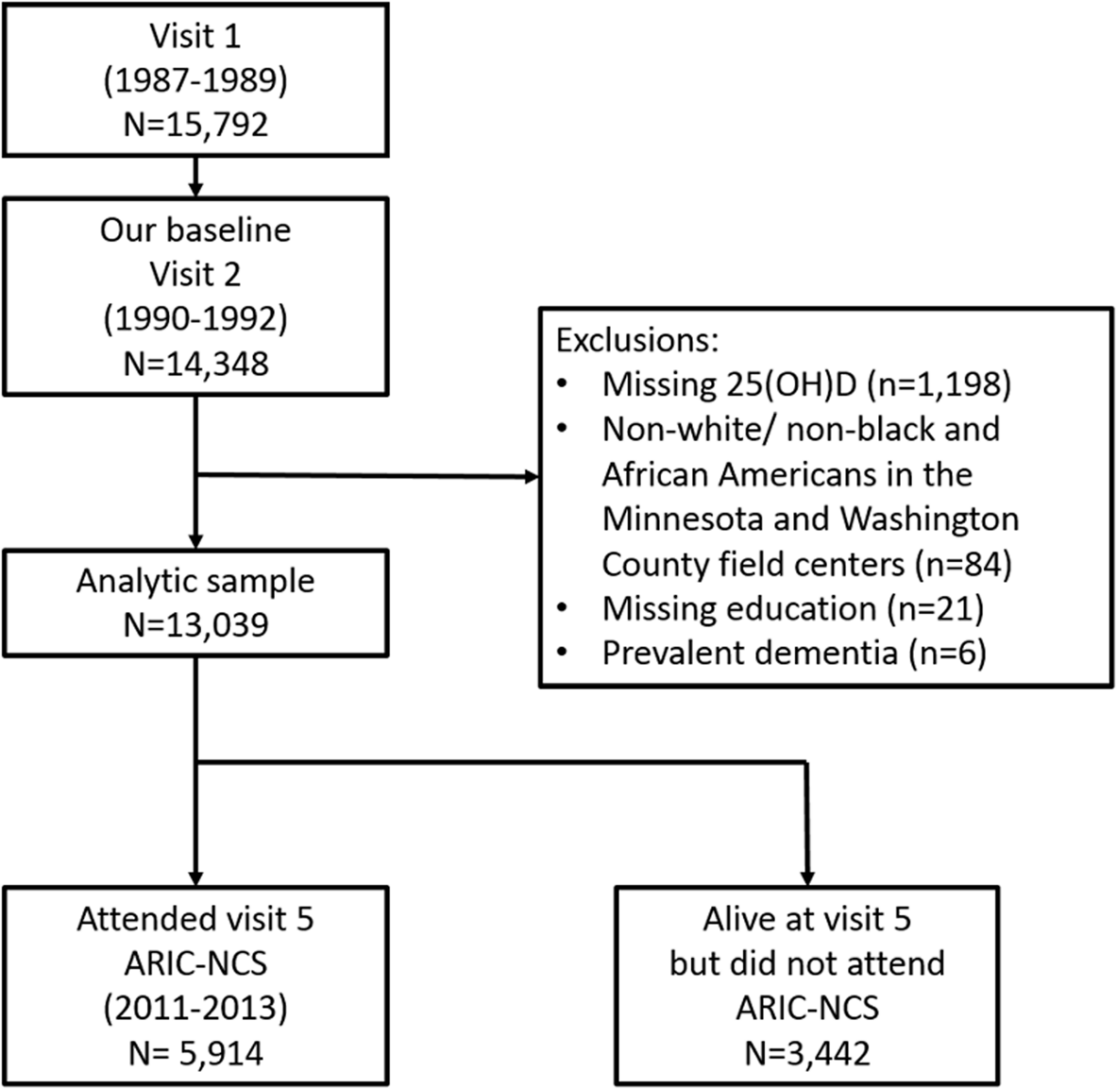
Participant Flow chart illustrating our exclusions

The present analysis includes all ARIC participants (*N* = 13,039) who had 25(OH) D measurement in mid-life at visit 2 (1990–1992) and were free from dementia at this visit. See Fig. 1 for exclusions. At ARIC visit 5 (2011–2013), all surviving participants were invited to participate in the ARIC Neurocognitive Study (ARIC-NCS) exam (*n* = 5914). In this present study, we examined the association of mid-life (visit 2) 25(OH) D levels with neuropsychological performance assessed at the ARIC-NCS study ~ 20-years later and with incident dementia occurring over this 20-year follow-up.

### Covariates of interest

Serum 25(OH) D was only measured at ARIC visit 2 for the whole cohort, which is why this visit is the baseline for the present analysis. The baseline characteristics of study participants were obtained at visit 2 from questionnaires, medication inventory, physical exam, and laboratory data, except as otherwise noted. Body mass index (BMI) was measured from height and weight. Systolic blood pressure (SBP) was calculated from the mean of the second and third measurements out of three obtained. Diabetes was defined as a fasting glucose ≥126 mg/dL or non-fasting glucose ≥200 mg/dL, a self-reported physician diagnosis of diabetes, or current use of hypoglycemic medication. Total and HDL-cholesterol were measured using standardized enzymatic assays [23]. The Chronic Kidney Disease Epidemiology Collaboration formula [24] was used to estimate glomerular filtration rate (eGFR). Serum intact parathyroid hormone (PTH) concentrations was measured using an Elecsys assay (Roche Diagnostics, Indianapolis, IN), and calcium and phosphate concentrations were measured by a colorimetric method through a Modular P-Chemistry Analyzer (Roche Diagnostics) [25].

### 25(OH) D measurement

Serum 25(OH) D concentrations were measured from fasting samples that had been collected at visit 2 (1990–1992) and stored at − 70 °C until measured in 2012–2013 using liquid chromatography-tandem high-sensitivity mass spectrometry (Waters Alliance e2795, Milford, MA, USA) [26]. Concentrations of serum/plasma 25(OH) D have previously been shown to have long-term stability when measured from frozen samples [27, 28]. The coefficients of variation were 20.8 and 6.9% for 25(OH)D_2_ and 25(OH)D3, respectively. Using blind duplicate samples, the intraclass correlation coefficients, calculated by the icc() function in the R package irr, were 0.96 (95% CI 0.95–0.96) for 25(OH)D_2_ and 0.91 (0.86–0.92) for 25(OH)D_3_. Total 25(OH) D concentration was calculated from the sum of 25(OH)D_2_ and 25(OH)D_3_. To convert 25(OH) D from ng/mL to nmol/L, multiply by 2.496.

### Outcome measures

Previously, in the ARIC cohort, we evaluated the association of 25(OH) D with cognitive decline using a global cognitive Z-score based on repeated measures of just 3 cognitive tests [7, 8], and no relationships were found. Our primary outcome for the present analysis is the association of mid-life 25(OH) D with the late-life performance on a more extensive battery of neuropsychological tests at the ARIC-NCS exam.

Neuropsychological testing at the ARIC-NCS were administered in a quiet room by trained examiners using standardized protocols [29, 30] and included the following cognitive domains: memory (Delayed Word Recall Test, Logical Memory Test Part I and II, Incidental Learning), language (Animal Naming, Boston Naming Test, Word Fluency Test), and processing speed and executive function (Trail Making Test A and B, Digital Symbol Substitution Test, Digit Span Backwards). Secondary outcomes assessed includes functional ability [Functional Activities Questionnaire (FAQ), Time to Walk 4 m, Short Physical Performance Battery (SPPB) score and grip strength], mental status [Mini-Mental State Examination (MMSE)], and depressive symptoms using the 11-item Center for Epidemiological Studies-Depression (CES-D). The (Additional file 1: Supplemental Methods) section provides more details about how these tests are performed and the normative scores for the ARIC cohort.

### Dementia ascertainment

Incident dementia cases in ARIC were adjudicated by an expert dementia classification committee comprised of eight clinicians including four physicians and four neuropsychologists [31]. Dementia was ascertained using a pre-determined algorithm, incorporating data from the cognitive tests performed at ARIC visits 2, 4, and 5, the full neuropsychological assessment performed at visit 5, participant interviews, informant (family member) interviews, and also hospital discharge codes and diagnostic codes from death certificates, as has been previously described in ARIC [31–35]. Each dementia case was adjudicated by a physician (either a geriatrician or neurologist) and a neuropsychologist, and in cases of disagreement a third clinician confirmed the diagnosis.

### Statistical analyses

We used seasonally-adjusted 25(OH) D concentrations as previously described in ARIC [26] and categorized them based on established clinical cut-points [36, 37] as deficient (< 20), intermediate (20- < 30), or sufficient (≥30 ng/mL). We also examined 25(OH) D continuously per standard deviation (SD) decrement. We used multivariable-adjusted linear regression models to examine the non-concurrent cross-sectional associations between mid-life 25(OH) D (at visit 2) and later-life neuropsychological and cognitive testing, functional ability, and depressive symptoms (at visit 5). Cox regression models were used for the incident dementia analysis. We verified that the proportionally hazards assumption was not violated by interacting 25(OH) D categories with log follow-up time; results were not significant.

We used progressively adjusted models. Model 1, our main model, adjusted for demographic, behavioral, and genetic factors: age (years), sex, race/center (MD-whites; MN-whites; NC-whites; NC-blacks; MS-blacks), education (<high school; high school or equivalent; college, graduate or professional school; assessed at visit 1), BMI (continuous, kg/m^2^), smoking status (current; former; never), alcohol consumption (current; former; never), physical activity (score range 1 to 5, using Baecke Physical Activity questionnaire [38] at visit 1), and APOE ε4 genotype. Model 2 further adjusted for cardiovascular disease risk factors: SBP, use of hypertension medication, total and HDL cholesterol (mg/dL), use of cholesterol medications, diabetes, prevalent coronary heart disease, prevalent stroke, and estimated glomerular filtration rate (eGFR, mL/min/1.73 m^2^). Model 3 additionally adjusted for biomarkers related to 25(OH) D metabolism: PTH (pg/mL), calcium (mg/dL), and phosphorus (mg/dL).

In our primary cognitive analyses, to account for missing data and loss of follow-up between visits 2 and 5, we imputed missing covariates at baseline (visit 2) and missing neurocognitive outcomes at the ARIC-NCS (visit 5) using multiple imputation by chained equation methods [39] (see Additional file 1: Table Se-1 for imputed numbers). This method of accounting for attrition is recommended by the ARIC-NCS working group and has been previously validated in the ARIC cohort [40]. However, in a sensitivity analysis, we also considered a “complete-case” analysis of only those participants who attended both visit 2 and visit 5 (2011–2013) (*n* = 5914). In another sensitivity analysis, we examined all participants who were not known to be deceased at the time of the ARIC-NCS visit (participants who came to the ARIC-NCS and those who did not attend but were alive). In our Cox models, we assessed for interactions by age, race/center, and sex. We considered *p*-values < 0.05 to be statistically significant and performed analyses using Stata Version 15 (StataCorp, College Station, TX).

## Results

### Baseline characteristics

Among the 13,039 participants, the mean (SD) age in mid-life was 57.4 (5.7) years and 25(OH) D concentration was 24.3 (8.6) ng/mL. Participants were 57% women, 24% black, and 33% had deficient, 44% intermediate, and 23% sufficient 25(OH) D concentrations. On average, participants with sufficient mid-life 25(OH) D were older, had lower BMI, SBP, total cholesterol, eGFR, PTH, and phosphate levels, and higher HDL-cholesterol and physical activity index compared to participants with deficient 25(OH) D concentrations. They were more likely to be white, current drinkers and on cholesterol lowering medications. They were less likely to be women, current smokers, on antihypertensive medications, have diabetes, or prevalent stroke (Table 1). The baseline characteristics of participants by attrition status is presented in Additional file 1: Table Se-2. Participants who attended the ARIC-NCS visit (at visit 5) tended to be younger, female, white, and had a more favorable cardio-metabolic profile at baseline than those who did not attend the ARIC-NCS visit.

**Table 1:** Baseline Characteristics of Participants, the ARIC Study (1990–1992)

Baseline characteristics	Overall	25(OH) D (ng/mL)		
		≥30 (Sufficient)	20- < 30 (Intermediate)	< 20 (Deficient)
N	13,039	3046	5759	4234
25(OH) D (ng/mL), ^b^ range	0.5–108.7	30–108.7	20- < 30	0.5- < 20
25(OH) D (ng/mL),^b^ mean (SD)	24.3 (8.6)	35.8 (5.9)	24.8 (2.8)	15.3 (3.5)
Age (years), mean (SD)	57.4 (5.7)	57.9 (5.7)	57.6 (5.7)	56.9 (5.7)
Women, *n* (%)	7384 (56.6%)	1490 (48.9%)	2998 (52.1%)	2896 (68.4%)
Race/Center, *n* (%)				
Minneapolis, MN Whites	3527 (27.1%)	981 (32.2%)	1721 (29.9%)	825 (19.5%)
Washington County, MD Whites	3373 (25.9%)	902 (29.6%)	1623 (28.2%)	848 (20%)
Forsyth County, NC Whites	2974 (22.8%)	961 (31.6%)	1400 (24.3%)	613 (14.5%)
Forsyth County, NC Blacks	344 (2.6%)	18 (0.6%)	94 (1.6%)	232 (5.5%)
Jackson, MS Blacks	2821 (21.6%)	184 (6%)	921 (16%)	1716 (40.5%)
Education, *n* (%) ^a^				
< High School	2785 (21.4%)	562 (18.5%)	1181 (20.5%)	1042 (24.6%)
High School, GED, or Vocational School	5451 (41.8%)	1358 (44.6%)	2392 (41.5%)	1701 (40.2%)
College, Graduate, or Professional School	4803 (36.8%)	1126 (37%)	2186 (38%)	1491 (35.2%)
BMI (kg/m^2^), mean (SD)	28.0 (5.4)	26.3 (4.2)	27.8 (5)	29.5 (6.3)
Physical activity index, mean (SD) ^a^	2.4 (0.8)	2.7 (0.8)	2.5 (0.8)	2.2 (0.7)
Current Smoker, *n* (%)	2857 (21.9%)	595 (19.5%)	1149 (20.0%)	1113 (26.3%)
Current Drinker, *n* (%)	7349 (56.4%)	1920 (63.0%)	3365 (58.4%)	2064 (48.8%)
Systolic Blood Pressure (mmHg), mean (SD)	121.5 (18.9)	119.2 (18.0)	120.8 (18.4)	124.1 (19.8)
Use of Hypertension Medications, *n* (%)	4282 (32.8%)	836 (27.5%)	1828 (31.7%)	1618 (38.2%)
Total Cholesterol (mg/dL), mean (SD)	210.1 (39.5)	209.3 (38)	210.3 (38.6)	210.4 (41.6)
HDL Cholesterol (mg/dL), mean (SD)	49.7 (16.8)	51.2 (17.9)	48.7 (16.1)	50.1 (16.8)
Use of Cholesterol Medications, *n* (%)	830 (6.4%)	225 (7.4%)	382 (6.6%)	223 (5.3%)
Diabetes, *n* (%)	1913 (14.7%)	272 (8.9%)	789 (13.7%)	852 (20.1%)
Prevalent Coronary Heart Disease, *n* (%)	753 (5.8%)	192 (6.3%)	333 (5.8%)	228 (5.4%)
Prevalent stroke, *n* (%)	252 (1.9%)	38 (1.3%)	116 (2%)	98 (2.3%)
eGFR (mL/min/1.73 m^2^), mean (SD)	96.3 (15.8)	92.7 (14)	95.5 (14.7)	99.9 (17.6)
Parathyroid hormone (pg/mL), mean (SD)	42.6 (23.9)	36.8 (12.7)	41.3 (18.6)	48.7 (33.3)
Calcium (mg/dL), mean (SD)	9.4 (0.4)	9.3 (0.4)	9.4 (0.4)	9.4 (0.5)
Phosphate (mg/dL), mean (SD)	3.5 (0.5)	3.5 (0.5)	3.5 (0.5)	3.6 (0.5)
Thyroid stimulating hormone (mIU/L), median (IQI)	1.8 (1.2–2.7)	1.9 (1.3–2.8)	1.8 (1.2–2.7)	1.7 (1.1–2.6)

Abbreviations: *ARIC* Atherosclerosis Risk in Communities; *eGFR* Estimated Glomerular Filtration Rate; *IQI* Interquartile interval

^a^ Measured at ARIC visit 1

^b^ To convert 25(OH) D from ng/mL to nmol/L, multiply by 2.496

### Neuropsychological test performance, functional ability, mental status, and depressive symptoms

**Fig. 2:**
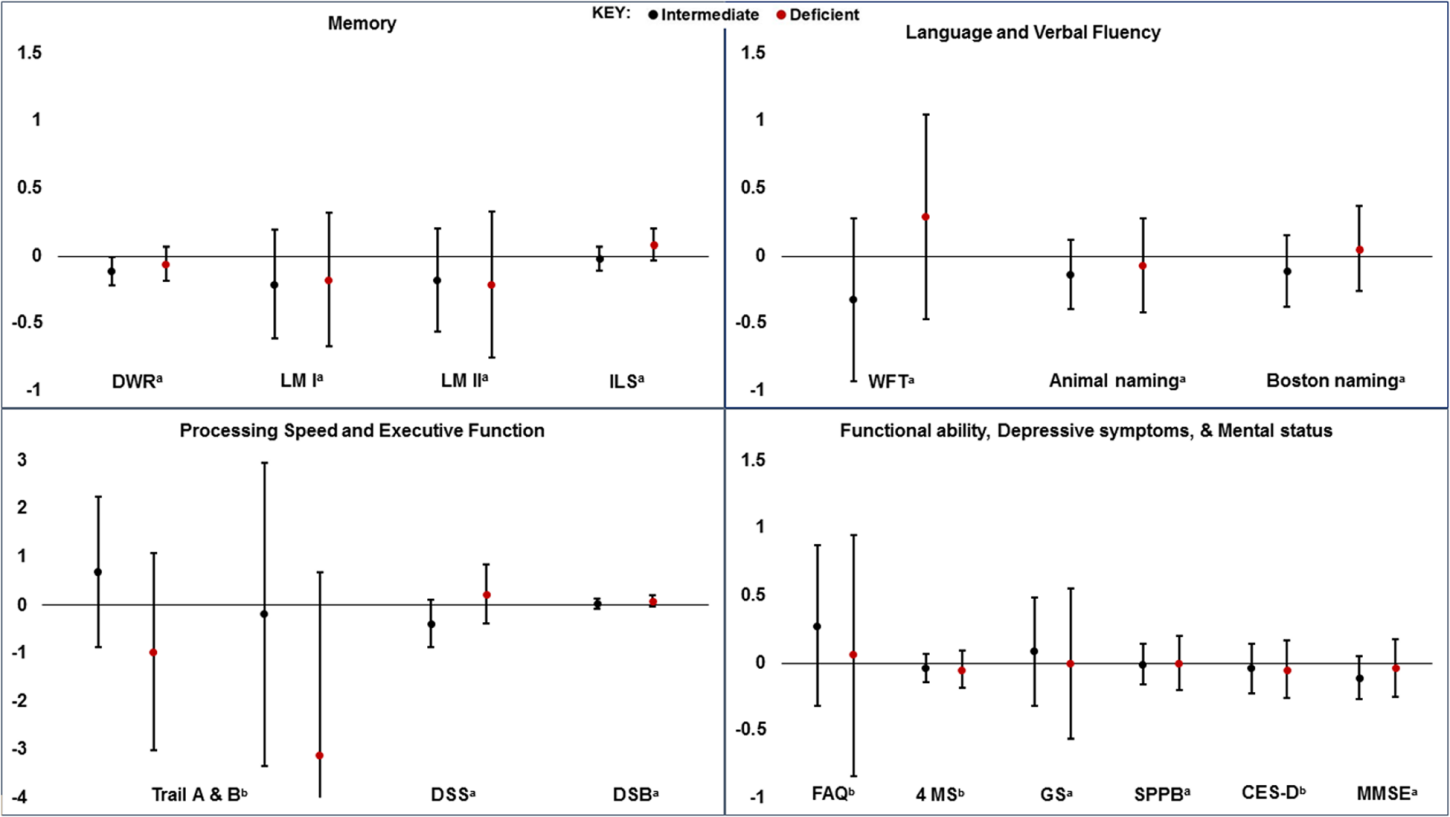
Association between mid-life 25(OH)D (1990-1992) and Later Life. Neuropsychological Test Performance (2011-2013): ARIC-NCS. DWR=Delayed word recall test; LM=Logical memory; IL=Incidental learning symbol; WFT=Word fluency test; DSS=Digit symbol substitution; DSB=Digit span backwards; FAQ=Functional activities questionnaire; 4 MS=Time to walk 4 meters; GS=Grip strength; SPPB=Short Physical Performance Battery; CES-D=Center for Epidemiologic. Studies Depression; MMSE=Mini-mental state exam. ^a^A higher value indicates a more favorable performance/measure; b a lower value indicates a more favorable performance/measure. Results were derived from multivariable linear regression models. Figure presents the adjusted difference in neuropsychological performance for intermediate 25(OH)D (20-29 ng/ml) [shown in black circles] and deficient vitamin D (<20 ng/ml) [shown in red circles], compared to sufficient 25(OH)D ≥30 ng/mL as reference. Models were adjusted for age, sex, race/center, educational, body mass index, smoking status, alcohol consumption, physical activity, and APOE ε. To covert 25(OH)D from ng/mL to nmol/L, multiply by 2.496

The mean (SD) age in late-life was 75.8 (5.3) years. 25(OH) D concentrations at mid-life were not associated with most of the neuropsychological testing outcomes (Fig. 2). In the domain of *memory*, we found intermediate concentrations of 25(OH) D to be associated with the Delayed Word Recall test in our main model but further adjustment for cardiovascular disease risk factors in model 2 yielded null results (Table 2). We found no association of 25(OH) D with outcomes in the domain of *language and verbal fluency* (Table 2). In the domain of *processing speed and executive function*, lower 25(OH) D per 1 SD decrement was associated with lower Trail B times and higher digit span backwards score (i.e. more favorable scores), which is in contrast to our hypotheses (Table 3). There was no significant associations of 25(OH) D with *functional ability, mental status, and depressive symptoms* 20-years after measurement of 25(OH) D (all *p* > 0.05, Table 4). In our sensitivity analyses, the results were also mostly null (and consistent with primary analysis) among only the participants who presented for the ARIC-NCS visit [*n* = 5914 (“complete-case” analysis); Additional file 1: Table Se-3] and among all the participants who were known to be alive at the ARIC-NCS visit (*n* = 9356; Additional file 1: Table Se-4).

**Table 2:** Adjusted Average Differences in Later Life Neuropsychological Test Performance Associated with Mid-Life 25(OH) D Concentrations: the ARIC Study ^a^

Neuropsychological outcomes	25(OH) D (ng/mL)			p-trend	Per 1 SD decrement in 25(OH) D ^b^
	≥30 (Sufficient)	20- < 30 (Intermediate)	< 20 (Deficient)		
N	3046	5759	4234		13,039
Memory					
Delayed word recall test ^c^					
Model 1 ^d^	0 (reference)	**−0.11 (− 0.22, − 0.01)**	− 0.06 (− 0.18, 0.07)	0.41	−0.01 (− 0.06, 0.03)
Model 2 ^e^	0 (reference)	−0.10 (− 0.20, 0.01)	−0.04 (− 0.17, 0.08)	0.58	− 0.01 (− 0.05, 0.04)
Model 3 ^f^	0 (reference)	− 0.10 (− 0.21, 0.003)	−0.06 (− 0.19, 0.07)	0.42	−0.01 (− 0.06, 0.03)
Logical memory I ^c^					
Model 1 ^d^	0 (reference)	−0.21 (− 0.61, 0.19)	−0.18 (− 0.67, 0.32)	0.51	−0.05 (− 0.24, 0.14)
Model 2 ^e^	0 (reference)	−0.22 (− 0.62, 0.18)	−0.19 (− 0.68, 0.31)	0.48	−0.06 (− 0.25, 0.13)
Model 3 ^f^	0 (reference)	−0.25 (− 0.65, 0.15)	−0.27 (− 0.76, 0.22)	0.29	−0.09 (− 0.28, 0.10)
Logical memory II ^c^					
Model 1 ^d^	0 (reference)	−0.18 (− 0.56, 0.20)	−0.21 (− 0.75, 0.33)	0.45	−0.04 (− 0.23, 0.16)
Model 2 ^e^	0 (reference)	−0.18 (− 0.57, 0.20)	−0.19 (− 0.73, 0.34)	0.49	− 0.03 (− 0.23, 0.17)
Model 3 ^f^	0 (reference)	−0.20 (− 0.58, 0.19)	−0.24 (− 0.76, 0.29)	0.38	−0.05 (− 0.25, 0.15)
Incidental learning symbol ^c^					
Model 1 ^d^	0 (reference)	−0.02 (− 0.11, 0.07)	0.08 (− 0.03, 0.20)	0.14	0.04 (− 0.01, 0.08)
Model 2 ^e^	0 (reference)	− 0.01 (− 0.10, 0.08)	0.10 (− 0.02, 0.22)	0.08	0.04 (− 0.001, 0.09)
Model 3 ^f^	0 (reference)	− 0.02 (− 0.11, 0.07)	0.07 (− 0.04, 0.19)	0.20	0.03 (− 0.01, 0.07)
Language and Verbal Fluency					
Word fluency ^c^					
Model 1 ^d^	0 (reference)	−0.32 (− 0.93, 0.28)	0.29 (− 0.47, 1.05)	0.40	0.17 (− 0.13, 0.46)
Model 2 ^e^	0 (reference)	−0.21 (− 0.82, 0.40)	0.50 (− 0.26, 1.27)	0.17	0.25 (− 0.05, 0.55)
Model 3 ^f^	0 (reference)	− 0.21 (− 0.83, 0.41)	0.48 (− 0.31, 1.26)	0.20	0.24 (− 0.08, 0.55)
Animal naming ^c^					
Model 1 ^d^	0 (reference)	−0.14 (− 0.39, 0.12)	−0.07 (− 0.42, 0.28)	0.72	0.001 (− 0.13, 0.14)
Model 2 ^e^	0 (reference)	−0.09 (− 0.35, 0.17)	0.03 (− 0.33, 0.38)	0.84	0.04 (− 0.10, 0.18)
Model 3 ^f^	0 (reference)	− 0.10 (− 0.36, 0.16)	0.01 (− 0.34, 0.35)	0.94	0.03 (− 0.10, 0.16)
Boston naming test score ^c^					
Model 1 ^d^	0 (reference)	−0.11 (− 0.38, 0.15)	0.05 (− 0.26, 0.37)	0.70	0.04 (− 0.08, 0.16)
Model 2 ^e^	0 (reference)	− 0.11 (− 0.38, 0.15)	0.07 (− 0.25, 0.38)	0.62	0.05 (− 0.07, 0.16)
Model 3 ^f^	0 (reference)	−0.09 (− 0.35, 0.17)	0.12 (− 0.19, 0.44)	0.39	0.07 (− 0.05, 0.19)

^a^ Results are presented as beta-coefficients (95% CI) derived from multivariable linear regression models. Sufficient 25(OH) D ≥ 30 ng/mL was the reference for the intermediate and deficient 25(OH) D categories. Data in bold text are statistically significant, *P* < .05

^b^ SD 25(OH) D = 8.4 ng/mL; To covert 25(OH) D from ng/mL to nmol/L, multiply by 2.496

^c^ A higher value indicates a more favorable performance/measure

^d^ Model 1: adjusted for age, sex, race/center, educational, body mass index, smoking status, alcohol consumption, physical activity, and APOE ε4 genotype

^e^ Model 2: Model 1 plus systolic blood pressure, use of hypertension medication, total and HDL cholesterol, use of cholesterol medications, diabetes, coronary heart disease, and estimated glomerular filtration rate

^f^ Model 3: Model 2 plus serum parathyroid hormone, calcium, and phosphorus concentrations

**Table 3:** Adjusted Average Differences in Later Life Neuropsychological Test Performance Associated with Mid-Life 25(OH) D Concentrations: the ARIC Study ^a^

Neuropsychological outcomes	25(OH) D (ng/mL)			p-trend	Per 1 SD decrement in 25(OH) D ^b^
	≥30 (Sufficient)	20- < 30 (Intermediate)	< 20 (Deficient)		
N	3046	5759	4234		13,039
Processing Speed and Executive Function					
Trail A ^c^					
Model 1 ^e^	0 (reference)	0.69 (− 0.88, 2.26)	− 0.97 (− 3.01, 1.07)	0.31	− 0.57 (− 1.40, 0.26)
Model 2 ^f^	0 (reference)	0.58 (− 1.00, 2.16)	− 1.30 (− 3.33, 0.73)	0.17	− 0.69 (− 1.52, 0.13)
Model 3 ^g^	0 (reference)	0.64 (− 0.92, 2.21)	−1.09 (− 3.08, 0.89)	0.24	− 0.60 (− 1.42, 0.22)
Trail B ^c^					
Model 1 ^e^	0 (reference)	− 0.19 (− 3.34, 2.96)	−3.12 (− 6.92, 0.67)	0.09	**−1.63 (− 3.11, − 0.16)**
Model 2 ^f^	0 (reference)	− 0.68 (− 3.87, 2.52)	**−4.00 (− 7.83, − 0.17)**	0.04	**−1.98 (− 3.49, − 0.46)**
Model 3 ^g^	0 (reference)	− 0.29 (− 3.43, 2.85)	−2.94 (− 6.69, 0.80)	0.11	**−1.55 (− 3.03, − 0.07)**
Digit symbol substitution ^d^					
Model 1 ^e^	0 (reference)	− 0.38 (− 0.88, 0.12)	0.23 (− 0.37, 0.84)	0.38	0.15 (− 0.09, 0.39)
Model 2 ^f^	0 (reference)	− 0.25 (− 0.75, 0.25)	0.47 (− 0.13, 1.08)	0.10	**0.25 (0.01, 0.49)**
Model 3 ^g^	0 (reference)	− 0.27 (− 0.77, 0.23)	0.39 (− 0.22, 1.00)	0.17	0.21 (− 0.03, 0.45)
Digit span backwards ^d^					
Model 1 ^e^	0 (reference)	0.03 (− 0.08, 0.13)	0.09 (− 0.03, 0.21)	0.13	**0.05 (0.01, 0.09)**
Model 2 ^f^	0 (reference)	0.04 (− 0.07, 0.14)	0.10 (− 0.02, 0.22)	0.09	**0.05 (0.01, 0.09)**
Model 3 ^g^	0 (reference)	0.03 (− 0.08, 0.14)	0.10 (− 0.02, 0.22)	0.11	**0.05 (0.01, 0.09)**

^a^ Results are presented as beta-coefficients (95% CI) derived from multivariable linear regression models. Sufficient 25(OH) D ≥ 30 ng/mL was the reference for the intermediate and deficient 25(OH) D categories. Data in bold text are statistically significant, *P* < .05

^b^ SD 25(OH) D = 8.4 ng/mL; To covert 25(OH) D from ng/mL to nmol/L, multiply by 2.496

^c^For Trail A and Trail B: a lower value indicates a more favorable performance/measure

^d^ For digit symbol substitution and digit span backwards: a higher value indicates a more favorable performance/measure

^e^ Model 1: adjusted for age, sex, race/center, educational, body mass index, smoking status, alcohol consumption, physical activity, and APOE ε4 genotype

^f^ Model 2: Model 1 plus systolic blood pressure, use of hypertension medication, total and HDL cholesterol, use of cholesterol medications, diabetes, coronary heart disease, and estimated glomerular filtration rate

^g^ Model 3: Model 2 plus serum parathyroid hormone, calcium, and phosphorus concentrations

**Table 4:** Adjusted Average Differences in Later Life Neuropsychological Test Performance Associated with Mid-Life 25(OH) D Concentrations: the ARIC Study ^a^

Neuropsychological outcomes	25(OH) D (ng/mL)			p-trend	Per 1 SD decrement in 25(OH) D ^b^
	≥30 (Sufficient)	20- < 30 (Intermediate)	< 20 (Deficient)		
N	3046	5759	4234		13,039
Functional Ability, Mental status, and Depressive symptoms					
Functional activities questionnaire (FAQ) score ^c^					
Model 1 ^e^	0 (reference)	0.27 (− 0.32, 0.87)	0.06 (− 0.84, 0.95)	0.93	−0.08 (− 0.47, 0.31)
Model 2 ^f^	0 (reference)	0.24 (−0.35, 0.84)	0.02 (− 0.86, 0.90)	0.99	− 0.09 (− 0.48, 0.29)
Model 3 ^g^	0 (reference)	0.29 (− 0.31, 0.89)	0.16 (− 0.73, 1.05)	0.75	− 0.04 (− 0.43, 0.35)
Time to walk 4 m (sec) ^d^					
Model 1 ^e^	0 (reference)	−0.04 (− 0.14, 0.07)	−0.05 (− 0.18, 0.09)	0.49	−0.03 (− 0.09, 0.02)
Model 2 f	0 (reference)	−0.05 (− 0.15, 0.05)	−0.07 (− 0.20, 0.06)	0.32	−0.04 (− 0.10, 0.01)
Model 3 ^g^	0 (reference)	−0.06 (− 0.16, 0.05)	−0.07 (− 0.21, 0.06)	0.27	−0.04 (− 0.10, 0.01)
Grip strength ^c^					
Model 1 ^e^	0 (reference)	0.09 (−0.32, 0.49)	−0.001 (− 0.56, 0.55)	0.98	0.03 (− 0.19, 0.25)
Model 2 ^f^	0 (reference)	0.12 (−0.28, 0.52)	0.07 (− 0.48, 0.61)	0.83	0.06 (− 0.16, 0.27)
Model 3 ^g^	0 (reference)	0.12 (−0.27, 0.52)	0.06 (−0.48, 0.60)	0.84	0.05 (−0.16, 0.26)
Short Physical Performance Battery (SPPB) ^c^					
Model 1 ^e^	0 (reference)	−0.01 (− 0.16, 0.14)	0.0003 (− 0.20, 0.20)	0.99	0.02 (− 0.06, 0.10)
Model 2 ^f^	0 (reference)	0.02 (−0.13, 0.17)	0.05 (− 0.15, 0.25)	0.61	0.04 (− 0.04, 0.12)
Model 3 ^g^	0 (reference)	0.02 (−0.12, 0.17)	0.05 (−0.15, 0.25)	0.63	0.04 (−0.04, 0.12)
Mini-mental state exam (MMSE) ^c^					
Model 1 ^e^	0 (reference)	−0.11 (− 0.27, 0.05)	−0.04 (− 0.25, 0.18)	0.77	0.002 (− 0.08, 0.08)
Model 2 ^f^	0 (reference)	−0.11 (− 0.27, 0.05)	−0.03 (− 0.24, 0.19)	0.85	0.01 (− 0.07, 0.08)
Model 3 ^g^	0 (reference)	−0.11 (− 0.27, 0.06)	−0.03 (− 0.25, 0.19)	0.81	0.002 (− 0.08, 0.09)
Depression score (CESD) ^d^					
Model 1 ^e^	0 (reference)	−0.04 (− 0.23, 0.14)	−0.05 (− 0.26, 0.17)	0.68	−0.04 (− 0.12, 0.05)
Model 2 ^f^	0 (reference)	−0.06 (− 0.25, 0.13)	−0.07 (− 0.29, 0.15)	0.53	−0.04 (− 0.13, 0.04)
Model 3 ^g^	0 (reference)	−0.07 (− 0.25, 0.12)	−0.09 (− 0.31, 0.13)	0.40	−0.05 (− 0.14, 0.03)

^a^ Results are presented as beta-coefficients (95% CI) derived from multivariable linear regression models. Sufficient 25(OH) D ≥ 30 ng/mL was the reference for the intermediate and deficient 25(OH) D categories

^b^ SD 25(OH) D = 8.4 ng/mL; To covert 25(OH) D from ng/mL to nmol/L, multiply by 2.496

^c^ For FAQ score, grip strength, SPPB, and MMSE: a higher value indicates a more favorable performance/measure

^d^ For time to walk 4 m and CESD: a lower value indicates a more favorable performance/measure

^e^ Model 1: adjusted for age, sex, race/center, educational, body mass index, smoking status, alcohol consumption, physical activity, and APOE ε4 genotype

^f^ Model 2: Model 1 plus systolic blood pressure, use of hypertension medication, total and HDL cholesterol, use of cholesterol medications, diabetes, coronary heart disease, and estimated glomerular filtration rate

^g^ Model 3: Model 2 plus serum parathyroid hormone, calcium, and phosphorus concentrations

### Dementia

There were 1323 incident cases of dementia over a median follow-up of 20 years (235,308 person-years). The unadjusted incidence rate (95% CI) per 1000 person-years were 4.83 (4.29, 5.44), 5.73 (5.28, 6.20), and 6.07 (5.54, 6.66) for sufficient, intermediate, and deficient concentrations of 25(OH) D, respectively. When compared to participants with sufficient 25(OH) D, the hazard ratios (95% CI) were 1.12 (0.97, 1.30) for intermediate and 1.26 (1.06, 1.49) for deficient 25(OH) D concentrations after adjustment for variables in our main model which included demographic, behavioral, and genetic factors, *p* = 0.01 for trend across categories (Table 5, model 1). Results remained statistically significant for trends across categories (*p* = 0.01) in our fully-adjusted model 3, with an increased risk for incident dementia (HR 1.24 [95% CI 1.05, 1.48] for participants with deficient 25(OH) D, compared to sufficient concentrations. There were no interactions by age, race, or sex (all *p* > 0.05).

**Table 5:** Associations ^a^ of Mid-Life (1990–1992) 25(OH) D Concentrations with Incident Dementia, the ARIC Study

	25(OH) D (ng/mL)			p-trend	Per 1 SD decrement in 25(OH) D ^c^
	≥30	20- < 30	< 20		
N	3046	5759	4234	–	13,039
Case, *n* (%)	271 (8.9%)	600 (10.4%)	452 (10.7%)	–	1323 (10.1%)
Incidence rate (95% CI) ^b^	4.83 (4.29, 5.44)	5.73 (5.28, 6.20)	6.07 (5.54, 6.66)	–	5.62 (5.33, 5.93)
Model 1 ^d^	Reference [1]	1.12 (0.97, 1.30)	**1.26 (1.06, 1.49)**	0.01	1.05 (0.98, 1.12)
Model 2 ^e^	Reference [1]	1.12 (0.97, 1.30)	**1.25 (1.05, 1.48)**	0.01	1.04 (0.98, 1.12)
Model 3 ^f^	Reference [1]	1.12 (0.97, 1.30)	**1.24 (1.05, 1.48)**	0.01	1.04 (0.98, 1.11)

^a^ Results presented as Hazard Ratios (95% Confidence Intervals). Data in bold text are statistically significant, *P* < .05

^b^ unadjusted and per 1000 person-years

^c^ SD 25(OH) D = 8.4 ng/mL;To covert 25(OH) D from ng/mL to nmol/L, multiply by 2.496

^d^ Model 1: adjusted for age, sex, race/center, educational, body mass index, smoking status, alcohol consumption, physical activity, and APOE ε4 genotype

^e^ Model 2: Model 1 plus systolic blood pressure, use of hypertension medication, total and HDL cholesterol, use of cholesterol medications, diabetes, coronary heart disease, and estimated glomerular filtration rate

^f^ Model 3: Model 2 plus serum parathyroid hormone, calcium, and phosphorus concentrations

## Discussion

In this community-based cohort, deficient 25(OH) D concentrations in mid-life were significantly associated with risk of incident dementia but were mostly not associated with late-life performance on neuropsychological testing, functional ability, or depression testing approximately 20-years later.

Low serum concentrations of 25(OH) D have previously been associated with stroke [26], coronary heart disease [26], and cardiovascular risk factors such as hypertension, diabetes, and lipids [23, 41, 42]. A growing body of evidence has also suggested a possible role of vitamin D in the development of dementia [1] and our findings appears to be consistent with some of such observational studies [5]. This may be due to the role of activated vitamin D in preserving healthy neurons and protection against known risk factors of dementia [1, 2, 5]. Despite this, vitamin D supplementation has not been shown to prevent dementia in prior randomized control trials, albeit some of these trials used lower doses of vitamin D supplements or studied patients with existing impaired cognition [43, 44]. It is also possible that vitamin D in the form of supplementation does not confer the same benefit as when obtained from natural sources such as diet and sunlight. Vitamin D supplementation, when combined with calcium supplements, may actually be associated with some harms such as hypercalcemia, hypercalcuria, kidney stones, and vascular events [19, 45–47]. However, a Mendelian randomization study did not find evidence of genetically-determined 25(OH) D concentrations to be causally related to cognitive function [48].

Using data from the ARIC cohort, we previously had found that 25(OH) D concentrations were not associated with 10-year or 20-year cognitive decline using a global Z-score based on repeated measures of just 3 cognitive tests [7, 8]. However, in a subset of the ARIC cohort (*n* = 1652) from the southern ARIC field centers (Jackson, MS and Forsyth County, NC) who were participating in the ARIC Brain MRI ancillary study, we found a suggestive association of lower 25(OH) D concentrations with hospitalized incident dementia (based on only ICD9 codes), but results were not statistically significant [7]. In that earlier study, comparing the lowest vs. highest race-specific tertiles of 25(OH) D, the hazard ratio for incident dementia for whites was 1.32 (95% CI 0.69, 2.55) and for blacks was 1.53 (0.84, 2.79)], but our analyses may have been underpowered with only 145 cases of hospitalized incident dementia [7].

In this extension of our prior work, our present study provides 1) a much larger sample size from ARIC (*N* = 13,039) from all 4 field centers (including the northern sites of Minnesota and Maryland), 2) a more extensive battery of neuropsychological and cognitive testing outcomes, 3) a longer follow-up period (20-years) and 4) a greater number of incident dementia cases (*N* = 1323) that have now been formally adjudicated. In the present analyses, the determination of incident dementia used a very formal algorithm that incorporated all of the available data from in-person testing spanning over 20-years, participant and informant interviews, and ICD9 codes from hospitalizations and death certificates that was then adjudicated by an expert clinician panel.

We now confirm a failure to find a consistent association of mid-life 25(OH) D with performance on a more extensive battery of neuropsychological, functional, and depressive symptoms testing administered 20-years later. In an exploratory model, we also examined more extreme vitamin D deficiency [comparing severe deficiency (< 5 ng/mL), moderate deficiency (5- < 10 ng/mL), and mild 25(OH) D deficiency (10- < 20 ng/mL) to adequate 25(OD) H concentrations (≥ 20 ng/mL)] with neuropsychological performance measures and findings were still largely null and consistent with primary analyses (results not shown).

However, in our current analysis, we did find an association with incident dementia, which warrants further explanation. Considering that some of these neuropsychological testing outcomes were used in the adjudication of dementia, our inability to find positive associations with most of these individual neuropsychological tests seems inconsistent with the positive association we found with incident dementia. Dementia affects the quality of life and may have prevented some participants from returning for the ARIC-NCS visit where all the neuropsychological, functional ability, and depression testing were done; whereas the diagnosis of incident dementia was made by both in-person visits and also by telephone interviews and hospitalization surveillance outside of the visit and included individuals who were unable or unwilling to attend the in-person visits. We did account for this attrition for the in-person visits with multiple imputation using methods previously validated in ARIC [40]; however some selection bias may have remained. It is possible that 25(OH) D is associated with dementia through other vascular mechanisms not captured by these neuropsychological and functional tests. Or it is possible that the association of 25(OH) D and incident dementia may be due to residual confounding in this observational study, despite our efforts to perform robust statistical adjustment for a number of key confounding factors.

The VITamin D and OmegA-3 TriaL (VITAL), which randomized over 20,000 community-dwelling adults aged ≥50 years to a higher dose of vitamin D supplementation (2000 IU/day) vs. placebo, will further evaluate the role of vitamin D supplementation for the prevention of cognitive decline [49]. Note that the VITAL trial recently reported that vitamin D supplementation did not reduce incident cardiovascular disease or cancer outcomes [50], but the cognitive outcomes have not yet been reported. The Study to Understand Vitamin D and Fall Reduction in You (STURDY) is an on-going randomized clinical trial investigating the role of vitamin D supplements (at doses ranging from 200 to 4000 IU/day) on the outcomes of functional measures (short physical performance battery, gait speed, and grip strength) and incident falls [16], which will also be informative when published.

In older adults, lower 25(OH) D concentrations have also been shown to be cross-sectionally associated with depressive symptoms [51, 52]. Tryptophan hydroxylase 2 (the catalyst which produces serotonin, a neurotransmitter implicated in the pathogenesis of depression, from tryptophan) has been found to be modulated by 25(OH) D [53]. However, a prior study of older adults (the Pro.V.A. Study) only found 25(OH) D to be cross-sectionally but not longitudinally associated with depressive symptoms [52]. In another analysis of older women enrolled in the Women’s Health Initiative Observational Study, higher vitamin D intake from food sources was associated with a lower risk of depression after 3 years; however supplementation with vitamin D (400 IU/day) in the Women’s Health Initiative randomized clinical trial was not associated with depression score [54]. In this current analysis from ARIC, we did not find any association of mid-life 25(OH) D with late-life depression score (assessed by CESD). The VITAL clinical trial mentioned above, evaluating a higher dose of vitamin D supplements (2000 IU/day) vs. placebo, will provide further understanding of the role of vitamin D for the prevention of depression in mid-life and older adults [55].

### Strengths and limitations

Our study has several limitations, which should be noted. First, we had only one measure of 25(OH) D in mid-life for the whole cohort (which we seasonally-adjusted); however 25(OH) D concentrations may vary over time and one-time measurements may not be reflective of concentrations at the time outcomes were ascertained. We also examined the use of vitamin D supplementation across visits (i.e. ARIC visits 2–5) and found little to no significant differences in proportion of participants who were on supplementation across baseline 25(OH) D groups at a given ARIC visit. Additionally, vitamin D supplement use was very infrequent at ARIC visit 2 (baseline for this analysis), at < 2%. Second, we imputed outcomes for a large number of participants not present at the ARIC-NCS visit. Nevertheless, “complete-case” results for participants present at the ARIC-NCS visit (visit 5) were very similar to those obtained from our imputation. We performed numerous linear regression models for a large battery of neuropsychological/functional tests and any significant associations may have been found by chance; however our findings for the neuropsychological/functional tests were largely null, so correcting for multiple testing would only further emphasize the null relationships noted. Other factors that might be associated with dementia (such as homocysteine, folate, B-vitamins, dietary intake of dairy products and fatty fish etc.) were not measured in ARIC at visit 2. (A food frequency questionnaire was administered only at ARIC visit 1 and 3). Thyroid stimulating hormone (TSH) was measured but it was not associated with 25(OH) D after adjustment for age, race, and sex.

Importantly though, our study has many strengths. In the well-characterized ARIC cohort, we were able to evaluate the association of mid-life 25(OH) D concentrations with a large battery of neuropsychological and functional tests conducted approximately 20-years later, as well as incident dementia outcomes over this same period. We measured 25(OH) D in mid-life when our participants were free from dementia and assessed outcomes in later life (~ 20 years), thereby limiting the effect of reverse causation, a problem which has plagued many other studies. We accounted for numerous potentially confounding lifestyle variables which have been found to be associated with serum 25(OH) D concentrations [56] and increased risk of dementia such as increasing age, black race, physical activity etc. as well as for the APOE ε4 genotype [57]. Individuals in poorer health states may be less likely to participate in outdoor physical activity and thus more likely to be 25(OH) D deficient. Nevertheless, despite robust adjustment, residual confounding may still explain associations see with incident dementia. Finally, our method of dementia ascertainment may be more comprehensive than other studies because in addition to review of medical and death certificate records, an in-person neuropsychological assessment, informant interview, and expert review were all used to adjudicate the cases of dementia [31–35].

## Conclusions

In conclusion, we found lower concentrations of 25(OH) D in mid-life were not associated with an extensive battery of neuropsychological, functional, and depression testing in late-life, but were associated with incident dementia in this biracial cohort. Although our long-term prospective design makes reverse causation unlikely, residual confounding may still explain associations seen. It is difficult to explain the association of serum 25(OH) D with dementia, given the lack of association of 25(OH) D concentrations with well-measured cognitive testing spanning 20 years [8]. Low serum concentrations of 25(OH) D may simply be a surrogate marker of a poorer health status. The VITAL interventional trial of vitamin D supplements (at 2000 IU/day) vs placebo failed to show a reduction in cancer or cardiovascular outcomes [50], but the cognitive and depression outcomes from VITAL have not yet been reported. Thus, whether the association with incident dementia is causal, or due to another process, is unclear and warrants further study in the on-going interventional trials.

## Supplementary information

**Additional file 1.** Supplemental Methods.

## Data Availability

The ARIC cohort participates in the National Heart, Lung, and Blood Institute’s Biologic Specimen and Data Repository (BioLINCC). The ARIC data are available upon request through BioLINCC (https://biolincc.nhlbi.nih.gov/studies/aric/).

## Abbreviations

25(OH)D: 25-hydroxyvitamin D
ARIC: Atherosclerosis Risk in Communities
ARIC-NCS: ARIC Neurocognitive Study
BMI: Body mass index
CDR: Clinical Dementia Rating
CES-D: Center for Epidemiological Studies-Depression
eGFR: estimated glomerular filtration rate
FAQ: Functional Activities Questionnaire
MMSE: Mini-mental state examination
PTH: Parathyroid hormone
SBP: Systolic blood pressure
SPPB: Short Physical Performance Battery
TICSm: Telephone Interview for Cognitive Status–Modified
VDR: Vitamin D receptors
VITAL: VITamin D and OmegA-3 TriaL
