# Supplementary material for: Mid-life serum Vitamin D concentrations were associated with incident dementia but not late-life neuropsychological performance in the Atherosclerosis Risk in Communities (ARIC) Study
Source: BMC Neurol. 2019 Oct 22;19:244. doi: 10.1186/s12883-019-1483-3 (PMC6805504; doi:10.1186/s12883-019-1483-3)
Supplement: Supplementary file 1 — Additional file 1. Supplemental Methods. [file 12883_2019_1483_MOESM1_ESM.docx]

**Supplemental Methods about the Neuropsychological Tests Performed**

*Memory*

The Delayed Word Recall Test is a test of verbal learning and recent memory where participants are given 10 common nouns to learn by using each word in one or two sentences. Participants were given 60 seconds to recall the 10 words after a five-minute delay. The score is the number of words correctly recalled [1, 2]. In ARIC-NCS, this score ranged from 0-10.

The Logical Memory Test is a test of immediate (Part I) and delayed (Part II) memory from the Wechsler Memory Scale-Revised. Two short stories (A and B) are presented, each containing 25 pieces of information. Free recall of the story is elicited immediately after each story is presented and after 30-minutes delay. The score for Part I is derived as the total number of story elements recalled immediately and the score for Part II is derived after free recall of each story after the 30-minutes delay [1, 2]. The scores ranged from 0-45.

The Incidental Learning Test is based on the Digit Symbol Substitution Test. Participants were asked to remember the symbols and corresponding digit–symbol pairs immediately after the Digit Symbol Substitution Test. The number of symbol pairs correctly recalled is scored [2]. This score ranged from 0 to 9.

*Language*

The Animal Naming is a test of semantic category fluency in which participants are asked to spontaneously generate words from a specific category (in our case, animals). Participants were allowed to name multiple words in the same subcategory (e.g., dog, poodle, dalmatian). The total number of animals generated within 60 seconds is the Animal Naming score [1, 2]. This score ranged from 1-39.

The Boston Naming Test assesses the participant’s ability to correctly name objects in a series of 30 line drawings. Participants were given 20 seconds to name each object. No clues are provided and the number of drawings correctly identified is scored [2]. This score ranged from 2-30.

The Word Fluency Test is a test of executive function and language which examines the ability of participants to spontaneously generate words excluding proper nouns beginning with “F”, “A”, and “S”. Participants were given 60 seconds for each of the letters. The score is the total number of acceptable words generated for the three letters [1, 2]. This score ranged from 0-80.

*Processing Speed and Executive Function*

The Trail Making Test A and B are tests of processing speed. In Test A, participants were asked to draw a line connecting circles numbered 1 to 25 that are randomly distributed on a page as fast as possible. In Test B, participants were asked to draw a line connecting the numbers 1 to 13 and the letters A to L that are randomly distributed on a page, alternating between numbers and letters. A maximum of 240 seconds is allotted for each test and the score is the time (seconds) taken to complete the task [1, 2]. Each test ranged from 0-240 seconds with a higher score reflecting worse performance.

The Digital Symbol Substitution Test is a test of processing speed and executive function where participants are asked to translate numbers to symbols using a key. The score is the total number of numbers correctly translated to symbols within 90-seconds and ranges from 0 to 93 [1, 2]. In ARIC-NCS, this score ranged from 2-80.

The Digit Span Backwards requires participants to read a series of numbers increasing in length from two to seven digits each. Participants were then asked to repeat each series backward having two trials for each digit span length [1, 2]. This score ranged from 0-12.

*Functional Ability*

The FAQ is a 10-item instrument which accesses participants ability to perform 10 common daily tasks [3]. This ranged from 0-30.

The Time to walk 4 meters was derived from the faster of two trials in which participants walked at a normal pace [4]. This ranged from 2-47.05 seconds.

The SPPB is a test of physical function in which participants were scored based on their performance on three tasks: balance (standing, semitandem, tandem stands held for up to 10 seconds each), usual gait speed over 4 meters, and time to rise from a seated position five times without using the arms. Based on population thresholds, each task was scored from 0 (worse) to 4 (best) and summed to create a composite score from 0 to 12 [4].

Grip strength was assessed twice in the participants preferred hand (usually the dominant) using the Jamar Hydraulic Hand Dynamometer. We used the better of the two trials for analysis [5]. This ranged from 0-90 kilograms of force.

*Mental Status*

MMSE is serves as a screening tool for the detection of cognitive impairment using 11 questions [6]. Scores range from 0 to 30 with higher scores indicating better performance. A prorated score was calculated from the total number of items completed for participants who did not complete the full MMSE due to reasons such as physical impairment [7].

*Depressive symptoms*

CES-D short form is an 11-item questionnaire assesses depressive symptoms [3]. Participants were asked to rate each item on a 3-point scale based on “how often you have felt this way during the past week.” scored 0 (Hardly ever or never) to 2 (Much or most of the time of). Higher scores indicated more severe depressive symptoms and ranges from 0 to 22. In ARIC-NCS, this ranged from 0-19.

1. Schneider AL, Sharrett AR, Gottesman RF, Coresh J, Coker L, Wruck L, Selnes OA, Deal J, Knopman D, Mosley TH: Normative data for 8 neuropsychological tests in older blacks and whites from the atherosclerosis risk in communities (ARIC) study. *Alzheimer Dis Assoc Disord* 2015, 29(1):32-44.

2. Rawlings AM, Bandeen-Roche K, Gross AL, Gottesman RF, Coker LH, Penman AD, Sharrett AR, Mosley TH: Factor structure of the ARIC-NCS Neuropsychological Battery: An evaluation of invariance across vascular factors and demographic characteristics. *Psychol Assess* 2016, 28(12):1674-1683.

3. Knopman DS, Gottesman RF, Sharrett AR, Wruck LM, Windham BG, Coker L, Schneider ALC, Hengrui S, Alonso A, Coresh J *et al*: Mild cognitive impairment and dementia prevalence: The Atherosclerosis Risk in Communities Neurocognitive Study. *Alzheimer's & Dementia : Diagnosis, Assessment & Disease Monitoring* 2016, 2:1-11.

4. Windham BG, Harrison KL, Lirette ST, Lutsey PL, Pompeii LA, Gabriel KP, Koton S, Steffen LM, Griswold ME, Mosley TH: Relationship Between Midlife Cardiovascular Health and Late-Life Physical Performance: The ARIC Study. *Journal of the American Geriatrics Society* 2017, 65(5):1012-1018.

5. Matsushita K, Ballew SH, Sang Y, Kalbaugh C, Loehr L, Hirsch AT, Tanaka H, Heiss G, Windham BG, Selvin E *et al*: Ankle-brachial index and physical function in older individuals: The Atherosclerosis Risk in Communities (ARIC) Study. *Atherosclerosis* 2017, 257:208-215.

6. Folstein MF, Folstein SE, McHugh PR: "Mini-mental state". A practical method for grading the cognitive state of patients for the clinician. *Journal of psychiatric research* 1975, 12(3):189-198.

7. Gottesman RF, Schneider AL, Zhou Y, Chen X, Green E, Gupta N, Knopman DS, Mintz A, Rahmim A, Sharrett AR *et al*: The ARIC-PET amyloid imaging study: Brain amyloid differences by age, race, sex, and APOE. *Neurology* 2016, 87(5):473-480.

**Table e-1.** Number of Participants with Missing Data that was Imputed.

| Variable | N |
| --- | --- |
| Covariates | - |
| Body mass index | 16 |
| Physical activity index | 46 |
| Total Cholesterol | 25 |
| HDL Cholesterol | 63 |
| Smoking status | 19 |
| Driving status | 17 |
| Systolic Blood Pressure | 1 |
| Use of Cholesterol Medications | 33 |
| Diabetes | 32 |
| Prevalent stroke | 23 |
| Parathyroid hormone | 135 |
| Calcium | 136 |
| Phosphate | 135 |
| APOE genotype | 391 |
| Outcomes | - |
| **Memory** | - |
| Delayed Word Recall | 6854 |
| Logical memory I | 7004 |
| Logical memory II | 7052 |
| Incidental learning symbol score | 7046 |
| **Language and Verbal Fluency** | - |
| Word fluency total score | 6873 |
| Animal naming score | 6797 |
| Boston naming test score | 6933 |
| **Processing Speed and Executive Function** | - |
| Trail A | 7055 |
| Trail B | 7571 |
| Digit symbol substitution | 6970 |
| Digit span backwards | 6973 |
| **Functional Ability, Depression and Mental status** | - |
| Functional Activities Questionnaire | 10302 |
| Time to walk 4 meters | 7253 |
| Grip strength | 7227 |
| Short Physical Performance Battery | 7198 |
| Mini-mental state exam | 6711 |
| Center for Epidemiologic Studies Depression | 6784 |

**Table e-2**. Baseline Characteristics of ARIC Study Participants (in 1990-1992) by Attrition Status at Visit 5 (2011-2013).

| Baseline characteristics | Overall | Alive and attended visit 5 | Alive and did not attend visit 5 | Dead prior to visit 5 |
| --- | --- | --- | --- | --- |
| N | 13,039 | 5,914 | 3,442 | 3,683 |
| 25(OH)D (ng/mL)^b^, mean (SD) | 24.3 (8.6) | 24.6 (8.4) | 24.5 (8.7) | 23.7 (8.7) |
| Age (years), mean (SD) | 57.4 (5.7) | 55.5 (5.1) | 57.8 (5.7) | 60.1 (5.5) |
| Women, n (%) | 7384 (56.6%) | 3549 (60%) | 2138 (62.1%) | 1697 (46.1%) |
| Race/Center, n (%) |  |  |  |  |
| Minneapolis, MN Whites | 3527 (27.1%) | 1783 (30.2%) | 901 (26.2%) | 843 (22.9%) |
| Washington County, MD Whites | 3373 (25.9%) | 1613 (27.3%) | 797 (23.2%) | 963 (26.2%) |
| Forsyth County, NC Whites | 2974 (22.8%) | 1208 (20.4%) | 963 (28.0%) | 803 (21.8%) |
| Forsyth County, NC Blacks | 344 (2.6%) | 88 (1.5%) | 138 (4.0%) | 118 (3.2%) |
| Jackson, MS Blacks | 2821 (21.6%) | 1222 (20.7%) | 643 (18.7%) | 956 (26.0%) |
| Education, n (%) ^a^ |  |  |  |  |
| <High School | 2785 (21.4%) | 847 (14.3%) | 808 (23.5%) | 1130 (30.7%) |
| High School, GED, or Vocational School | 5451 (41.8%) | 2503 (42.3%) | 1497 (43.5%) | 1451 (39.4%) |
| College, Graduate, or Professional School | 4803 (36.8%) | 2564 (43.4%) | 1137 (33.0%) | 1102 (29.9%) |
| BMI (kg/m^2^), mean (SD) | 28.0 (5.4) | 27.7 (5.1) | 28.2 (5.4) | 28.3 (5.8) |
| Physical activity index, mean (SD) ^a^ | 2.4 (0.8) | 2.5 (0.8) | 2.4 (0.8) | 2.4 (0.8) |
| Current Smoker, n (%) | 2857 (21.9%) | 948 (16.0%) | 704 (20.5%) | 1205 (32.7%) |
| Current Drinker, n (%) | 7349 (56.4%) | 3593 (60.8%) | 1854 (53.9%) | 1902 (51.6%) |
| Systolic Blood Pressure (mmHg), mean (SD) | 121.5 (18.9) | 117.8 (16.7) | 121.8 (18.1) | 127.0 (21.4) |
| Use of Hypertension Medications, n (%) | 4282 (32.8%) | 1451 (24.5%) | 1145 (33.3%) | 1686 (45.8%) |
| Total Cholesterol (mg/dL), mean (SD) | 210.1 (39.5) | 207.6 (37.1) | 213.7 (39.6) | 210.6 (42.7) |
| HDL Cholesterol (mg/dL), mean (SD) | 49.7 (16.8) | 51.2 (16.7) | 50.2 (16.7) | 47.0 (16.7) |
| Use of Cholesterol Medications, n (%) | 830 (6.4%) | 304 (5.1%) | 232 (6.7%) | 294 (8%) |
| Diabetes, n (%) | 1913 (14.7%) | 509 (8.6%) | 469 (13.6%) | 935 (25.4%) |
| Prevalent Coronary Heart Disease, n (%) | 753 (5.8%) | 153 (2.6%) | 135 (3.9%) | 465 (12.6%) |
| Prevalent stroke, n (%) | 252 (1.9%) | 47 (0.8%) | 40 (1.2%) | 165 (4.5%) |
| eGFR (mL/min/1.73 m^2^), mean (SD) | 96.3 (15.8) | 98.1 (14) | 96.9 (14.6) | 92.8 (18.8) |
| Parathyroid hormone (pg/mL), mean (SD) | 42.6 (23.9) | 41.8 (16.5) | 42.1 (16.5) | 44.5 (36.5) |
| Calcium (mg/dL), mean (SD) | 9.4 (0.4) | 9.3 (0.4) | 9.4 (0.4) | 9.4 (0.5) |
| Phosphate (mg/dL), mean (SD) | 3.5 (0.5) | 3.5 (0.5) | 3.5 (0.5) | 3.5 (0.5) |

^a^ Measured at ARIC Visit 1 (1987-1989).

^b^To covert 25(OH)D from ng/mL to nmol/L, multiply by 2.496.

**Table e-3.** Adjusted Average Differences ^a^ (95% Confidence Intervals) in Later Life Neuropsychological Test Performance (2011-2013) Associated with Mid-Life (1990-1992) 25(OH)D Concentrations among Participants who Presented for the ARIC-NCS

| Variables |  | 25(OH)D (ng/mL) | | p-trend | Per 1 SD decrement  in 25(OH)D |
| --- | --- | --- | --- | --- | --- |
| N = 5,914 | ≥30  (Sufficient) | 20-<30  (Intermediate) | <20  (Deficient) |  |  |
| **Memory** |  |  |  |  |  |
| Delayed word recall test ^b^ |  |  |  |  |  |
| Model 1 | 0 (reference) | -0.11 (-0.23, 0.01) | -0.02 (-0.16, 0.12) | 0.82 | -0.004 (-0.06, 0.05) |
| Model 2 | 0 (reference) | -0.10 (-0.22, 0.02) | -0.01 (-0.15, 0.13) | 0.90 | -0.002 (-0.05, 0.05) |
| Model 3 | 0 (reference) | -0.10 (-0.22, 0.02) | -0.01 (-0.16, 0.13) | 0.87 | -0.003 (-0.06, 0.05) |
| Logical memory I ^b^ |  |  |  |  |  |
| Model 1 | 0 (reference) | -0.13 (-0.59, 0.34) | -0.10 (-0.64, 0.45) | 0.74 | -0.03 (-0.23, 0.18) |
| Model 2 | 0 (reference) | -0.14 (-0.60, 0.32) | -0.13 (-0.68, 0.41) | 0.64 | -0.05 (-0.26, 0.16) |
| Model 3 | 0 (reference) | -0.13 (-0.60, 0.33) | -0.16 (-0.72, 0.39) | 0.56 | -0.06 (-0.27, 0.15) |
| Logical memory II ^b^ |  |  |  |  |  |
| Model 1 | 0 (reference) | -0.12 (-0.60, 0.36) | -0.16 (-0.73, 0.40) | 0.57 | 0.01 (-0.21, 0.22) |
| Model 2 | 0 (reference) | -0.13 (-0.61, 0.36) | -0.18 (-0.75, 0.39) | 0.54 | -0.005 (-0.22, 0.21) |
| Model 3 | 0 (reference) | -0.12 (-0.60, 0.36) | -0.19 (-0.77, 0.38) | 0.51 | -0.01 (-0.23, 0.21) |
| Incidental learning symbol ^b^ |  |  |  |  |  |
| Model 1 | 0 (reference) | -0.03 (-0.14, 0.07) | 0.11 (-0.02, 0.24) | 0.09 | 0.04 (-0.01, 0.09) |
| Model 2 | 0 (reference) | -0.03 (-0.14, 0.08) | 0.11 (-0.02, 0.24) | 0.08 | 0.04 (-0.01, 0.09) |
| Model 3 | 0 (reference) | -0.03 (-0.14, 0.08) | 0.10 (-0.03, 0.23) | 0.12 | 0.03 (-0.02, 0.08) |
| **Language and Verbal Fluency** |  |  |  |  |  |
| Word fluency ^b^ |  |  |  |  |  |
| Model 1 | 0 (reference) | -0.33 (-1.06, 0.40) | 0.41 (-0.45, 1.28) | 0.33 | 0.24 (-0.09, 0.56) |
| Model 2 | 0 (reference) | -0.28 (-1.01, 0.45) | 0.53 (-0.33, 1.40) | 0.21 | 0.28 (-0.05, 0.61) |
| Model 3 | 0 (reference) | -0.25 (-0.99, 0.48) | 0.52 (-0.35, 1.40) | 0.23 | 0.28 (-0.06, 0.61) |
| Animal naming ^b^ |  |  |  |  |  |
| Model 1 | 0 (reference) | -0.13 (-0.43, 0.17) | -0.08 (-0.43, 0.27) | 0.67 | 0.02 (-0.11, 0.15) |
| Model 2 | 0 (reference) | -0.12 (-0.42, 0.18) | -0.03 (-0.39, 0.32) | 0.87 | 0.03 (-0.10, 0.16) |
| Model 3 | 0 (reference) | -0.11 (-0.41, 0.19) | -0.03 (-0.38, 0.33) | 0.88 | 0.04 (-0.10, 0.17) |
| Boston naming test score ^b^ |  |  |  |  |  |
| Model 1 | 0 (reference) | -0.12 (-0.39, 0.14) | 0.20 (-0.12, 0.52) | 0.20 | 0.09 (-0.03, 0.21) |
| Model 2 | 0 (reference) | -0.13 (-0.40, 0.14) | 0.18 (-0.13, 0.50) | 0.24 | 0.08 (-0.04, 0.20) |
| Model 3 | 0 (reference) | -0.10 (-0.37, 0.17) | 0.24 (-0.08, 0.56) | 0.13 | 0.11 (-0.01, 0.23) |
| **Processing Speed and Executive Function** | |  |  |  |  |
| Trail a ^c^ |  |  |  |  |  |
| Model 1 | 0 (reference) | 1.32 (-0.52, 3.17) | -0.99 (-3.22, 1.23) | 0.35 | -0.56 (-1.40, 0.27) |
| Model 2 | 0 (reference) | 1.39 (-0.44, 3.23) | -0.92 (-3.15, 1.30) | 0.38 | -0.52 (-1.35, 0.32) |
| Model 3 | 0 (reference) | 1.33 (-0.51, 3.18) | -0.93 (-3.19, 1.33) | 0.39 | -0.51 (-1.37, 0.35) |
| Trail b ^c^ |  |  |  |  |  |
| Model 1 | 0 (reference) | -0.20 (-3.74, 3.34) | -2.69 (-6.93, 1.56) | 0.21 | **-1.72 (-3.31, -0.12)** |
| Model 2 | 0 (reference) | -0.30 (-3.83, 3.23) | -2.86 (-7.10, 1.37) | 0.18 | **-1.74 (-3.32, -0.15)** |
| Model 3 | 0 (reference) | -0.26 (-3.79, 3.28) | -2.47 (-6.75, 1.82) | 0.25 | -1.58 (-3.20, 0.04) |
| Digit symbol substitution ^b^ |  |  |  |  |  |
| Model 1 | 0 (reference) | -0.46 (-1.09, 0.16) | 0.38 (-0.36, 1.11) | 0.29 | 0.21 (-0.07, 0.49) |
| Model 2 | 0 (reference) | -0.43 (-1.04, 0.19) | 0.46 (-0.27, 1.20) | 0.19 | 0.24 (-0.04, 0.52) |
| Model 3 | 0 (reference) | -0.39 (-1.01, 0.23) | 0.47 (-0.28, 1.21) | 0.20 | 0.24 (-0.05, 0.52) |
| Digit span backwards ^b^ |  |  |  |  |  |
| Model 1 | 0 (reference) | 0.03 (-0.10, 0.15) | 0.07 (-0.08, 0.21) | 0.38 | 0.05 (-0.01, 0.10) |
| Model 2 | 0 (reference) | 0.03 (-0.09, 0.16) | 0.07 (-0.08, 0.21) | 0.35 | 0.05 (-0.01, 0.10) |
| Model 3 | 0 (reference) | 0.03 (-0.09, 0.16) | 0.07 (-0.08, 0.22) | 0.37 | 0.05 (-0.01, 0.10) |
| **Functional Ability, Mental status, and Depressive symptoms** | | |  |  |  |
| FAQ score ^c^ |  |  |  |  |  |
| Model 1 | 0 (reference) | 0.35 (-0.32, 1.02) | 0.18 (-0.60, 0.97) | 0.66 | -0.02 (-0.35, 0.31) |
| Model 2 | 0 (reference) | 0.34 (-0.33, 1.01) | 0.20 (-0.60, 0.99) | 0.64 | -0.01 (-0.35, 0.32) |
| Model 3 | 0 (reference) | 0.34 (-0.33, 1.01) | 0.23 (-0.58, 1.04) | 0.58 | 0.005 (-0.34, 0.35) |
| Time to walk 4 meters (sec) ^c^ |  |  |  |  |  |
| Model 1 | 0 (reference) | -0.05 (-0.16, 0.05) | -0.005 (-0.13, 0.12) | 0.96 | -0.03 (-0.07, 0.02) |
| Model 2 | 0 (reference) | -0.05 (-0.16, 0.05) | -0.01 (-0.14, 0.12) | 0.92 | -0.03 (-0.07, 0.02) |
| Model 3 | 0 (reference) | -0.07 (-0.18, 0.04) | -0.03 (-0.16, 0.10) | 0.65 | -0.04 (-0.09, 0.01) |
| Grip strength ^b^ |  |  |  |  |  |
| Model 1 | 0 (reference) | 0.20 (-0.29, 0.69) | -0.14 (-0.71, 0.44) | 0.62 | -0.004 (-0.22, 0.21) |
| Model 2 | 0 (reference) | 0.19 (-0.30, 0.68) | -0.15 (-0.73, 0.42) | 0.58 | -0.01 (-0.23, 0.21) |
| Model 3 | 0 (reference) | 0.22 (-0.27, 0.71) | -0.12 (-0.69, 0.46) | 0.68 | 0.005 (-0.21, 0.22) |
| SPPB ^b^ |  |  |  |  |  |
| Model 1 | 0 (reference) | -0.01 (-0.17, 0.14) | -0.08 (-0.27, 0.11) | 0.40 | 0.001 (-0.07, 0.07) |
| Model 2 | 0 (reference) | -0.01 (-0.16, 0.14) | -0.07 (-0.25, 0.12) | 0.48 | 0.01 (-0.06, 0.07) |
| Model 3 | 0 (reference) | 0.01 (-0.14, 0.16) | -0.04 (-0.23, 0.15) | 0.67 | 0.02 (-0.05, 0.09) |
| Mini-mental state exam ^b^ |  |  |  |  |  |
| Model 1 | 0 (reference) | -0.18 (-0.35, 0.001) | -0.07 (-0.28, 0.14) | 0.57 | -0.01 (-0.09, 0.07) |
| Model 2 | 0 (reference) | **-0.18 (-0.36, -0.003)** | -0.07 (-0.28, 0.14) | 0.52 | -0.02 (-0.10, 0.06) |
| Model 3 | 0 (reference) | -0.17 (-0.35, 0.01) | -0.06 (-0.27, 0.16) | 0.64 | -0.01 (-0.09, 0.07) |
| Depression score (CESD) ^c^ |  |  |  |  |  |
| Model 1 | 0 (reference) | -0.09 (-0.29, 0.10) | -0.06 (-0.29, 0.17) | 0.62 | -0.06 (-0.14, 0.03) |
| Model 2 | 0 (reference) | -0.10 (-0.30, 0.09) | -0.07 (-0.30, 0.16) | 0.57 | -0.06 (-0.15, 0.03) |
| Model 3 | 0 (reference) | -0.11 (-0.30, 0.09) | -0.09 (-0.32, 0.14) | 0.46 | -0.07 (-0.16, 0.02) |

CESD = Center for Epidemiologic Studies Depression; FAQ = Functional activities questionnaire; SPPB = Short Physical Performance Battery

^a^ Results were derived from multivariable linear regression models. Sufficient 25(OH)D ≥30 ng/mL was the reference for the intermediate and deficient 25(OH)D categories. Data in bold text are statistically significant, P < .05

^b^ a higher value indicates a more favorable performance/measure

^c^ a lower value indicates a more favorable performance/measure

SD 25(OH)D = 8.4 ng/mL

Model 1: adjusted for age, sex, race/center, educational, body mass index, smoking status, alcohol consumption, physical activity, and APOE ε4 genotype

Model 2: Model 1 plus systolic blood pressure, use of hypertension medication, total and HDL cholesterol, use of cholesterol medications, diabetes, coronary heart disease, and estimated glomerular filtration rate

Model 3: Model 2 plus serum parathyroid hormone, calcium, and phosphorus concentrations.

**Table e-4**. Adjusted Average Differences ^a^ (95% Confidence Intervals) in Later Life Neuropsychological Test Performance (2011-2013) Associated with Mid-Life (1990-1992) 25(OH)D Concentrations among Participants Alive During the ARIC-NCS

| Variables | 25(OH)D (ng/mL) | | | p-trend | Per 1 SD decrement in  25(OH)D |
| --- | --- | --- | --- | --- | --- |
| N = 9,356 | ≥30  (Sufficient) | 20-<30  (Intermediate) | <20  (Deficient) |  |  |
| **Memory** |  |  |  |  |  |
| Delayed word recall test ^b^ |  |  |  |  |  |
| Model 1 | 0 (reference) | **-0.13 (-0.24, -0.02)** | -0.02 (-0.15, 0.11) | 0.79 | -0.01 (-0.06, 0.04) |
| Model 2 | 0 (reference) | **-0.12 (-0.23, -0.01)** | -0.01 (-0.14, 0.12) | 0.90 | -0.002 (-0.05, 0.05) |
| Model 3 | 0 (reference) | **-0.12 (-0.23, -0.01)** | -0.02 (-0.15, 0.11) | 0.82 | -0.01 (-0.06, 0.05) |
| Logical memory I ^b^ |  |  |  |  |  |
| Model 1 | 0 (reference) | -0.26 (-0.68, 0.16) | -0.12 (-0.64, 0.40) | 0.66 | -0.04 (-0.24, 0.16) |
| Model 2 | 0 (reference) | -0.28 (-0.70, 0.15) | -0.14 (-0.66, 0.38) | 0.63 | -0.05 (-0.25, 0.15) |
| Model 3 | 0 (reference) | -0.29 (-0.71, 0.14) | -0.19 (-0.71, 0.33) | 0.48 | -0.08 (-0.28, 0.13) |
| Logical memory II ^b^ |  |  |  |  |  |
| Model 1 | 0 (reference) | -0.22 (-0.64, 0.20) | -0.13 (-0.69, 0.44) | 0.67 | -0.01 (-0.22, 0.21) |
| Model 2 | 0 (reference) | -0.23 (-0.65, 0.20) | -0.12 (-0.68, 0.44) | 0.70 | -0.01 (-0.22, 0.21) |
| Model 3 | 0 (reference) | -0.23 (-0.66, 0.20) | -0.15 (-0.72, 0.42) | 0.62 | -0.02 (-0.24, 0.20) |
| Incidental learning symbol ^b^ |  |  |  |  |  |
| Model 1 | 0 (reference) | -0.04 (-0.14, 0.05) | 0.09 (-0.04, 0.22) | 0.15 | 0.03 (-0.01, 0.08) |
| Model 2 | 0 (reference) | -0.04 (-0.14, 0.06) | 0.10 (-0.03, 0.23) | 0.11 | 0.04 (-0.01, 0.08) |
| Model 3 | 0 (reference) | -0.04 (-0.14, 0.06) | 0.09 (-0.04, 0.21) | 0.18 | 0.03 (-0.02, 0.08) |
| **Language and Verbal Fluency** |  |  |  |  |  |
| Word fluency ^b^ |  |  |  |  |  |
| Model 1 | 0 (reference) | -0.33 (-0.99, 0.32) | 0.50 (-0.28, 1.28) | 0.19 | 0.22 (-0.08, 0.52) |
| Model 2 | 0 (reference) | -0.25 (-0.91, 0.41) | 0.65 (-0.13, 1.43) | 0.09 | 0.28 (-0.02, 0.58) |
| Model 3 | 0 (reference) | -0.24 (-0.91, 0.43) | 0.63 (-0.18, 1.44) | 0.12 | 0.27 (-0.05, 0.59) |
| Animal naming ^b^ |  |  |  |  |  |
| Model 1 | 0 (reference) | -0.16 (-0.45, 0.14) | -0.04 (-0.42, 0.35) | 0.87 | 0.01 (-0.14, 0.16) |
| Model 2 | 0 (reference) | -0.13 (-0.42, 0.17) | 0.03 (-0.36, 0.42) | 0.85 | 0.03 (-0.12, 0.19) |
| Model 3 | 0 (reference) | -0.12 (-0.42, 0.17) | 0.03 (-0.35, 0.41) | 0.86 | 0.03 (-0.12, 0.19) |
| Boston naming test score ^b^ |  |  |  |  |  |
| Model 1 | 0 (reference) | -0.14 (-0.40, 0.12) | 0.19 (-0.15, 0.52) | 0.25 | 0.08 (-0.04, 0.20) |
| Model 2 | 0 (reference) | -0.14 (-0.40, 0.11) | 0.18 (-0.15, 0.51) | 0.26 | 0.08 (-0.05, 0.20) |
| Model 3 | 0 (reference) | -0.12 (-0.38, 0.14) | 0.23 (-0.10, 0.56) | 0.15 | 0.10 (-0.02, 0.22) |
| **Processing Speed and Executive Function** |  |  |  |  |  |
| Trail a ^c^ |  |  |  |  |  |
| Model 1 | 0 (reference) | 1.17 (-0.67, 3.00) | -1.18 (-3.40, 1.04) | 0.27 | -0.58 (-1.44, 0.28) |
| Model 2 | 0 (reference) | 1.20 (-0.63, 3.03) | -1.24 (-3.44, 0.96) | 0.24 | -0.58 (-1.43, 0.27) |
| Model 3 | 0 (reference) | 1.20 (-0.63, 3.04) | -1.15 (-3.34, 1.03) | 0.28 | -0.54 (-1.40, 0.32) |
| Trail b ^c^ |  |  |  |  |  |
| Model 1 | 0 (reference) | 0.33 (-2.95, 3.60) | -2.61 (-6.73, 1.51) | 0.20 | -1.42 (-2.92, 0.07) |
| Model 2 | 0 (reference) | 0.10 (-3.19, 3.39) | -3.10 (-7.25, 1.06) | 0.14 | **-1.58 (-3.10, -0.07)** |
| Model 3 | 0 (reference) | 0.28 (-3.01, 3.58) | -2.50 (-6.68, 1.68) | 0.23 | -1.35 (-2.86, 0.17) |
| Digit symbol substitution ^b^ |  |  |  |  |  |
| Model 1 | 0 (reference) | -0.47 (-1.04, 0.10) | 0.30 (-0.36, 0.96) | 0.34 | 0.14 (-0.11, 0.40) |
| Model 2 | 0 (reference) | -0.39 (-0.96, 0.17) | 0.45 (-0.21, 1.11) | 0.16 | 0.20 (-0.05, 0.45) |
| Model 3 | 0 (reference) | -0.38 (-0.96, 0.19) | 0.42 (-0.25, 1.10) | 0.20 | 0.19 (-0.07, 0.44) |
| Digit span backwards ^b^ |  |  |  |  |  |
| Model 1 | 0 (reference) | 0.02 (-0.08, 0.13) | 0.09 (-0.04, 0.22) | 0.15 | **0.05 (0.002, 0.10)** |
| Model 2 | 0 (reference) | 0.03 (-0.08, 0.13) | 0.10 (-0.03, 0.23) | 0.12 | **0.05 (0.004, 0.10)** |
| Model 3 | 0 (reference) | 0.02 (-0.08, 0.13) | 0.10 (-0.03, 0.23) | 0.14 | **0.05 (0.002, 0.10)** |
| **Functional Ability, Mental status, and Depressive symptoms** | | |  |  |  |
| FAQ score ^c^ |  |  |  |  |  |
| Model 1 | 0 (reference) | 0.38 (-0.25, 1.02) | 0.03 (-0.82, 0.88) | 0.97 | -0.07 (-0.44, 0.30) |
| Model 2 | 0 (reference) | 0.38 (-0.26, 1.02) | 0.03 (-0.81, 0.87) | 0.97 | -0.07 (-0.44, 0.30) |
| Model 3 | 0 (reference) | 0.39 (-0.25, 1.04) | 0.10 (-0.78, 0.97) | 0.84 | -0.04 (-0.43, 0.35) |
| Time to walk 4 meters (sec) ^c^ |  |  |  |  |  |
| Model 1 | 0 (reference) | -0.02 (-0.12, 0.08) | 0.003 (-0.12, 0.13) | 0.95 | -0.02 (-0.06, 0.03) |
| Model 2 | 0 (reference) | -0.02 (-0.13, 0.08) | -0.01 (-0.13, 0.12) | 0.90 | -0.02 (-0.07, 0.03) |
| Model 3 | 0 (reference) | -0.04 (-0.14, 0.07) | -0.03 (-0.15, 0.10) | 0.67 | -0.03 (-0.08, 0.02) |
| Grip strength ^b^ |  |  |  |  |  |
| Model 1 | 0 (reference) | 0.07 (-0.40, 0.53) | -0.14 (-0.66, 0.38) | 0.59 | -0.02 (-0.22, 0.19) |
| Model 2 | 0 (reference) | 0.07 (-0.40, 0.54) | -0.12 (-0.64, 0.40) | 0.64 | -0.01 (-0.22, 0.19) |
| Model 3 | 0 (reference) | 0.09 (-0.37, 0.55) | -0.10 (-0.62, 0.43) | 0.71 | -0.002 (-0.20, 0.20) |
| SPPB ^b^ |  |  |  |  |  |
| Model 1 | 0 (reference) | -0.05 (-0.20, 0.10) | -0.09 (-0.27, 0.10) | 0.35 | -0.01 (-0.08, 0.06) |
| Model 2 | 0 (reference) | -0.03 (-0.18, 0.11) | -0.06 (-0.24, 0.12) | 0.53 | -0.004 (-0.07, 0.06) |
| Model 3 | 0 (reference) | -0.02 (-0.17, 0.13) | -0.04 (-0.22, 0.15) | 0.69 | 0.005 (-0.07, 0.08) |
| Mini-mental state exam ^b^ |  |  |  |  |  |
| Model 1 | 0 (reference) | -0.17 (-0.34, 0.004) | -0.02 (-0.24, 0.21) | 0.90 | 0.004 (-0.08, 0.09) |
| Model 2 | 0 (reference) | -0.17 (-0.35, -0.004) | -0.02 (-0.25, 0.20) | 0.88 | 0.001 (-0.08, 0.09) |
| Model 3 | 0 (reference) | -0.17 (-0.34, 0.005) | -0.01 (-0.24, 0.21) | 0.94 | 0.004 (-0.08, 0.09) |
| Depression score (CESD) ^c^ |  |  |  |  |  |
| Model 1 | 0 (reference) | -0.04 (-0.22, 0.15) | -0.03 (-0.25, 0.19) | 0.77 | -0.03 (-0.11, 0.05) |
| Model 2 | 0 (reference) | -0.05 (-0.23, 0.13) | -0.05 (-0.27, 0.17) | 0.67 | -0.04 (-0.12, 0.05) |
| Model 3 | 0 (reference) | -0.05 (-0.23, 0.13) | -0.07 (-0.30, 0.15) | 0.54 | -0.05 (-0.13, 0.04) |

CESD = Center for Epidemiologic Studies Depression; FAQ = Functional activities questionnaire; SPPB = Short Physical Performance Battery

^a^ Results were derived from multivariable linear regression models. Sufficient 25(OH)D ≥30 ng/mL was the reference for the intermediate and deficient 25(OH)D categories. Data in bold text are statistically significant, P < .05

^b^ a higher value indicates a more favorable performance/measure

^c^ a lower value indicates a more favorable performance/measure

SD 25(OH)D = 8.4 ng/mL

Model 1: adjusted for age, sex, race/center, educational, body mass index, smoking status, alcohol consumption, physical activity, and APOE ε4 genotype

Model 2: Model 1 plus systolic blood pressure, use of hypertension medication, total and HDL cholesterol, use of cholesterol medications, diabetes, coronary heart disease, and estimated glomerular filtration rate

Model 3: Model 2 plus serum parathyroid hormone, calcium, and phosphorus concentrations.
